# Management of Invasive Fungal Infections in Patients Undergoing Allogeneic Hematopoietic Stem Cell Transplantation: The Turin Experience

**DOI:** 10.3389/fcimb.2021.805514

**Published:** 2022-01-07

**Authors:** Alessandro Busca, Natascia Cinatti, Jessica Gill, Roberto Passera, Chiara Maria Dellacasa, Luisa Giaccone, Irene Dogliotti, Sara Manetta, Silvia Corcione, Francesco Giuseppe De Rosa

**Affiliations:** ^1^ Department of Oncology, SSD Trapianto Allogenico di Cellule Staminali, Azienda Ospedaliera Universitaria (A.O.U.) Città della Salute e della Scienza di Torino, Turin, Italy; ^2^ Department of Medical Sciences, Division of Internal Medicine, University of Turin, Turin, Italy; ^3^ Department of Molecular Biotechnology and Health Sciences, Division of Hematology, University of Turin, Turin, Italy; ^4^ Department of Medical Sciences, Division of Nuclear Medicine, Azienda Ospedaliera Universitaria (A.O.U.) Città della Salute e della Scienza di Torino, Turin, Italy; ^5^ Department of Medical Sciences, Division of Infectious Diseases, Azienda Ospedaliera Universitaria (A.O.U.) Città della Salute e della Scienza di Torino, University of Turin, Turin, Italy

**Keywords:** invasive fungal infections, allogeneic hematopoietic stem cell transplantation, immunocompromised host, antifungal prophylaxis, bronchoalveolar lavage

## Abstract

**Background:**

Allogeneic hematopoietic stem cell transplant (allo-HSCT) recipients are exposed to an increased risk of invasive fungal infections (IFIs) due to neutropenia, immunosuppressive treatments, graft-versus-host disease (GvHD) and incomplete immune reconstitution. Although clinical benefit from antifungal prophylaxis has been demonstrated, IFIs remain a leading cause of morbidity and mortality in these patients. In the last decades, attention has also been focused on potential risk factors for IFI to tailor an antifungal prevention strategy based on risk stratification.

**Aim of the Study:**

This retrospective single-center study aimed to assess the epidemiology and the prognostic factors of IFI in a large cohort of allo-HSCT patients.

**Methods:**

Between January 2004 and December 2020, 563 patients with hematological malignancies received an allo-HSCT at the Stem Cell Transplant Unit in Turin: 191 patients (34%) received grafts from a matched sibling donor, 284 (50.5%) from a matched unrelated donor, and 87 (15.5%) from an haploidentical family member. The graft source was peripheral blood in 81.5% of the patients. Our policy for antifungal prophylaxis included fluconazole in matched related and unrelated donors, while micafungin was administered in patients receiving haploidentical transplant. According to this practice, fluconazole was administered in 441 patients (79.6%) and micafungin in 62 (11.2%), while only 9 patients received mold-active prophylaxis. Galactomannan testing was routinely performed twice a week; patients with persisting fever unresponsive to broad spectrum antibiotics were evaluated with lung high-resolution computed tomography (HRCT) scan. In case of imaging suggestive of IFI, bronchoalveolar lavage (BAL) was performed whenever feasible.

**Statistical Analysis:**

Only probable/proven IFI (PP-IFI) occurring during the first 12 months after transplant have been evaluated. IFIs were classified as probable or proven according to the new revised European Organization for Research and Treatment of Cancer (EORTC)/Mycoses Study Group (MSG) consensus criteria. Multivariate competing risk regression, binary logistic, and proportional hazard models were performed to identify risk factors for PP-IFI.

**Results:**

A total of 58 PP-IFIs (n = 47 probable; n = 11 proven) occurred in our patients resulting in a cumulative incidence of 4.1%, 8.1%, and 9.6% at 30, 180, and 365 days, respectively. Molds were the predominant agents (n = 50 *Aspergillus*; n = 1 *Mucor*), followed by invasive candidemia (n = 5 non-*albicans Candida*; n = 1 *Candida albicans*; n = 1 *Trichosporon*). Lung was the most frequent site involved in patients with mold infections (47/51, 92.2%). Median time from HSCT to IFI was 98.44 days (0–365 days). Only 34.5% of patients with IFI were neutropenic at the time of infection. The presence of IFI had a significant impact on overall survival at 1 year (IFI, 32.8% vs. non-IFI, 54.6%; p < 0.001). IFI-related mortality rate was 20.7% in the overall population, 17% in patients with probable IFI, and 36% in patients with proven IFI. Multivariate competing risk regression revealed that donor type was the factor significantly associated to the risk of IFI [subdistribution hazard ratio (SDHR), 1.91, IC 1.13–3.20; *p* = 0.015]. BAL was informative in a consistent number of cases (36/57, 63.2%) leading to the identification of fungal (21), bacterial (4), viral (3), and polymicrobial (8) infections. Overall, 79 patients (14%) received a diagnostic-driven treatment, and 63 patients (11.2%) received a fever-driven treatment. Liposomal amphoteric B was the drug used in the majority of patients receiving diagnostic-driven therapy (30/79, 38%), while caspofungin was administered more frequently in patients who received a fever-driven strategy (27/63, 42.9%).

**Conclusion:**

According to our experience, a non-mold active prophylaxis in patients undergoing allo-HSCT is feasible when combined with an intensive diagnostic work-up including CT scan and BAL. BAL performed at the onset of the disease may provide informative results in most patients. A diagnostic-driven treatment strategy may contribute to limit the use of costly antifungal therapies.

## Introduction

Hematopoietic stem cell transplantation (HSCT) has proven to be a curative treatment strategy for patients with many malignant and non-malignant hematological disorders, including leukemia, lymphomas, and aplastic anemia, and indications are still expanding (Gyurkocza et al., 2010; Copelan et al., 2018).

HSCT is a procedure that restores stem cells that have been destroyed by a preparative regimen including chemotherapy with or without total body irradiation delivered before stem cell infusion to optimize tumor cell kill and, in the case of allogeneic HSCT, immunosuppress the recipient to prevent graft rejection (Gagelmann and Kröger, 2021). In addition, allogeneic HSCT recipients receive immunosuppressive agents, namely, calcineurin inhibitors, for a prolonged period of time after transplant to mitigate the graft-versus-host reaction (Zeiser and Blazar, 2017; Martinez-Cibrian et al., 2020). According to these considerations, patients undergoing HSCT are at elevated risk for severe life-threatening infections (Sahin et al., 2016; Neofytos, 2019). Indeed, invasive fungal infections (IFIs) represent one of the major limiting factors for the successful outcome of patients receiving allogeneic HSCT (Bhatti et al., 2006). As a consequence, efforts to optimize the managements of IFI in hematological patients have assumed a great relevance (Rahi et al., 2021).

The use of antifungal prophylaxis is extremely diffused among hematological patients and allogeneic HSCT recipients, in accordance with the recommendations of the most authoritative guidelines (Girmenia et al., 2014; Mellinghoff et al., 2017; Maertens et al., 2018); however, several drawbacks have emerged over the last few years, such as the occurrence of side effects, the emergence of resistance, and the overuse of antifungals (Busca and Pagano, 2016; Schmiedel and Zimmerli, 2016). The advent of new diagnostic tools including HRCT and non-culture-based microbiological (NCBM) assays have increased significantly the accuracy in the diagnosis of IFI in hematological patients and HSCT recipients (Arendrup et al., 2012; Marchetti et al., 2012; Ruhnke et al., 2018). The potential advantage of a diagnostic-driven approach incorporating imaging, biomarkers, and early antifungal therapy may be considered as a more refined strategy over an empirical treatment based on the presence of persistent fever unresponsive to broad spectrum antibiotics (De Kort et al., 2019). In this respect, several trials have evaluated the feasibility of a pre-emptive strategy; however, the introduction of mold-active prophylaxis with NCBM methods and the definition of the most appropriate antifungal treatment are still matters of debate (Cordonnier et al., 2014; Fung et al., 2015).

The aim of the present study was to investigate whether the management of HSCT recipients at risk for IFI may be optimized with azole-based antifungal prophylaxis in conjunction with an aggressive diagnostic work-up.

The study is further implemented by the analysis of risk factors that might be potentially useful to tailor an appropriate treatment for HSCT patients with IFI.

## Materials and Methods

### Study Design and Data Collection

This retrospective cohort study included all consecutive adult patients who underwent an allogeneic HSCT for the treatment of a hematological malignancy at the Stem Cell Transplant Center AOU Città della Salute e della Scienza of Torino, between January 2004 and December 2020.

Indications for allogeneic HSCT were hematological malignant diseases and included acute myeloid leukemia (AML), myelodysplastic syndromes (MDS), acute lymphoblastic leukemia (ALL), lymphomas, chronic myeloid leukemia (CML), and multiple myeloma (MM). Written consent for transplant procedures and for the use of medical records for research purposes was obtained from all patients.

Medical chart and electronic records were reviewed retrospectively, and follow-up data were obtained until April 2021.

The exclusion criteria included transplant for non-hematological malignancies or for solid tumors. Patients receiving multiple allo-HSCT during the study period were censored at the time of second or third allo-HSCT, and data were collected independently for each single transplant

We considered only proven or probably IFI (PP-IFI) occurring within the first year of transplantation. In case of multiple fungal infections, only the first episode was considered in the analysis.

All procedures performed in the present study were in accordance with the ethical standards of the Institutional Review Board of the Città della Salute e della Scienza Hospital of Torino, Torino, Italy, according to the Declaration of Helsinki.

### Transplant Procedure

Patients were prepared for transplant either with myeloablative or reduced intensity/non-myeloablative conditionings according to their clinical conditions and HCT-CI. Reduced intensity/non-myeloablative regimens were usually preferred in patients over 55 years of age and/or in presence of pre-transplant comorbidities. By the European Blood and Marrow Transplant Group (EBMT) criteria, myeloablative conditionings had to contain a total busulfan dose >6.4 mg/kg i.v., or a cyclophosphamide dose >120 mg/kg (or >60 mg/kg if in combination with other drugs), or a melphalan dose >140 mg/m^2^, or a total body irradiation dose >6 Gy. Regimens with lower doses were defined as reduced intensity/non-myeloablative conditionings.

Graft-versus-host disease (GvHD) prophylaxis regimens included cyclosporin (CSA) and short-course methotrexate or CSA combined with micophenolate mofetil (MMF). *In vivo* T-cell depletion with thymoglobulin (ATG) was used in transplants from unrelated donors, mainly as 5–7 mg/kg divided into two or three doses. Patients transplanted from haploidentical donors received tacrolimus, MMF, and post-transplant cyclophosphamide as described by Luznik et al. (2001).

### Screening, Diagnostic Procedures for IFI, and Antifungal Practices

All patients candidate to allograft were cared in rooms with high-efficiency particulate air (HEPA) filter until engraftment was achieved and received antifungal prophylaxis.

Matched related and unrelated donor transplant recipients received fluconazole from day 0 to day +75 after HSCT or posaconazole if GvHD occurred; haploidentical transplant received micafungin until discharge, then fluconazole until day +75.

All patients were screened for invasive aspergillosis using serum galactomannan before HSCT and twice weekly after HSCT until their discharge and then during the follow-up until day +120.

In case of fever, standard procedures were performed including at least two sets of blood cultures at two different sites and serum β-D-glucan antigen test.

Lung high-resolution computed tomography (HRCT) was performed within the third day of persisting fever unresponsive to broad spectrum antibiotics or in case of respiratory symptoms. Bronchoscopy with bronchoalveolar lavage (BAL) was performed whenever feasible if HRCT was positive.

Galactomannan antigen testing was performed using the PlateliaTM Aspergillus Ag kit (BIO-RAD ^®^ Laboratories, Marnes, La Couquette, France) and was considered positive with a single value of ≥1.0 in serum or BAL fluid or with both values of ≥0.7 in serum and ≥0.8 in BAL fluid, according to new revised EORTC/MSG consensus criteria (Donnelly et al., 2020).

All fungal isolates growing in culture were identified as species according to their micromorphological characteristics and sequencing of their informative DNA target.

Broad spectrum antibiotic treatment was given on the first day of neutropenic fever.

Antifungal treatment was started on the basis of the diagnostic work-up in probable and proven fungal infections (diagnostic-driven treatment) or as empiric treatment in possible IFIs (fever-driven treatment).

### Definitions

We considered three different risk periods for fungal infection after allo-HSCT, defined as pre-engraftment phase (from day 0 to day +40), early post-engraftment phase (from days +41 to +100), and late post-engraftment phase (beyond day +100) (Carreras et al., 2019).

Neutropenia at the time of infection was defined as an absolute neutrophil count (ANC) <0.5 × 10^9^/L. If a white blood cell count was lacking, the latest available blood count within the week preceding was used. Neutrophil engraftment was defined as the first of three consecutive days with neutrophils >0.5 × 10^9^/L following allo-HSCT (Carreras et al., 2019).

IFIs were classified as probable or proven according to the new revised definition of the European Organization for Research and Treatment of Cancer (EORTC)/Mycoses Study Group (MSG) Consensus Group (Donnelly et al., 2020): proven disease required mycological documentation from a normally sterile site, while probable disease was supported by host factor, clinical features, and mycological evidence, like a positive galactomannan assay.

We considered the date of infection diagnosis as the day on which first positive diagnostic test was performed.

Primary antifungal therapy was defined as the first-line antifungal treatment administered after diagnosis.

Transplant risk for each patient was calculated using the hematopoietic cell transplantation-specific comorbidity index (HSCT-CI) (Sorror et al., 2005) and the revised Disease Risk Index (r-DRI) (Armand et al., 2014), respectively, for the comorbidity and the underlying disease impact.

Death was classified as IFI-related mortality if fungal infection disease was judged to be unresolved at the time of death and no alternative cause of death was identified by the treating physician nor from medical record review.

Both acute GvHD and chronic GvHD were diagnosed on the basis of clinical symptoms and/or biopsies according to standard criteria (Lee et al., 2002). Severity of chronic GvHD was assessed according to National Institutes of Health criteria and graded as mild, moderate, or severe (Filipovich et al., 2005; Jagasia et al., 2015).

## Statistical Analysis

The primary endpoint was the cumulative incidence (CI) for IFI (main event); competing event was the relapse/death without IFI, while alive patients were censored at the date of last contact (April 2021). The CI function was compared across groups by the Gray test, while the competing risks regression model was used to estimate the role of risk factors on IFI occurrence by the Fine–Gray test. The following covariates were tested as IFI occurrence determinants: recipient age (≥60 vs. 40–60 vs. <40 years), recipient gender (male vs. female), diagnosis (AML vs. other hematological malignancies), disease status at transplant (advanced vs. early disease), Sorror Comorbidity Index (HCT-CI, ≥3 vs. 0–2), donor type (haploidentical vs. MUD vs. MRD), Disease Risk Index (DRI, high/very high vs. intermediate vs. low), stem cell source (bone marrow vs. peripheral blood), conditioning regimen (reduced intensity vs. myeloablative), median CD34 doses infused/recipient weight (≥7.0 × 10^6^/kg vs. <7.0 × 10^6^/kg), median CD3 doses infused/recipient weight (≥2.5 × 10^8^/kg vs. <2.5 × 10^8^/kg), antifungal prophylaxis (micafungin vs. fluconazole), anti-thymocyte globulin (ATG) administration (yes vs. no), acute (grade II–IV vs. 0–I), and chronic GvHD (moderate and severe vs. no and mild) occurrence and median duration of neutropenia (≥16 vs. <16 days).

The secondary endpoint was the overall survival (OS), defined as the time from transplant to death from any cause. Survival curves were estimated by the Kaplan–Meier method and compared across groups by the log-rank test. The effect on OS of the same covariates set was analyzed by the Cox proportional hazards model, comparing the two arms by the Wald test and calculating 95% confidence intervals.

Patient characteristics were tested using the Fisher’s exact test for categorical variables and the Mann–Whitney and Kruskal–Wallis tests for continuous ones; continuous variables were described as median [interquartile range (IQR)]. All reported *p*-values were obtained by the two-sided exact method, at the conventional 5% significance level. Data were analyzed as of September 2021 by R 4.1.0 (R Foundation for Statistical Computing, Vienna-A, http://www.R-project.org).

## Results

### Patients’ Characteristics

The study included 523 patients receiving 563 allogeneic transplants: 36 patients had two, and 4 patients had three transplants. Three patients had a history of a previous allo-HSCT performed prior to inclusion period or in another center. Patients’ baseline characteristics are presented in **Table 1**.

**Table 1:** Main patients’ and transplants’ characteristics of the whole cohort and stratified by IFI.

Characteristics	All patients	IFI neg	IFI pos
Number of patients	563	504	58
Age at transplant, median (range), years	48 (18–70)	48 (18–70)	49 (22–67)
*Gender*			
Male	307 (54.5%)	275 (54.6%)	32 (55.2%)
Female	256 (45.5%)	229 (45.4%)	26 (44.8%)
*Underlying disease*			
AML/MDS	319 (56.7%)	283 (56.2%)	35 (60.3%)
ALL	84 (14.9%)	76 (15.1%)	8 (13.8%)
HL/NHL	101 (17.9%)	92 (18.3%)	9 (15.5%)
MPN/LMMC	49 (8.7%)	44 (8.7%)	5 (8.6%)
MM	10 (1.8%)	9 (1.8%)	1 (1.7%)
*HCT-CI*			
Low/intermediate (0–2)	236 (72.6%)	220 (73.8%)	15 (57.7%)
High (≥3)	89 (27.4%)	78 (26.2%)	11 (42.3%)
*DRI*			
Low	61 (11.3%)	56 (11.5%)	5 (9.6%)
Intermediate	367 (67.8%)	330 (67.8%)	35 (67.3%)
High	92 (17.0%)	83 (17.0%)	9 (17.3%)
Very high	21 (3.9%)	18 (3.7%)	3 (5.8%)
*Disease status at transplant*			
CR	282 (50.4%)	256 (50.9%)	25 (44.6%)
CR2	96 (17.1%)	84 (16.7%)	12 (21.4%)
PIF/relapse	182 (32.5%)	163 (32.4%)	19 (33.9%)
*Donor type*			
MSD	191 (34%)	182 (36.1%)	9 (15.8%)
MUD	284 (50.5%)	251 (49.8%)	33 (57. 9%)
Haploidentical^a^	87 (15.5%)	71 (14.1%)	15 (26.3%)
*Stem cell source*			
PBSC	458 (81.5%)	83 (16.5%)	12 (20.7%)
BM	96 (17.1%)	416 (82.5%)	43 (74.1%)
CB	8 (1.4%)	5 (1%)	3 (5.2%)
Number of CD34+ cells infused, median (IQR), x 10^6^/kg	7.0 (5.4–9.1)	7.1 (5.5–9.1)	6.4 (4.8–9.1)
Number of CD3+ cells infused, median (IQR), x 10^8^/kg	2.5 (1.5–3.3)	2.5 (1.5–3.4)	2.3 (1.2–3.1)
*Conditioning regimen*			
MAC	390 (69.4%)	355 (70.4%)	34 (59.6%)
RIC	172 (30.6%)	149 (29.6%)	23 (40.4%)
*GvHD prophylaxis*			
ATG	280 (50.4%)	251 (50.3%)	24 (42.9%)
other	276 (49.6%)	248 (49.7%)	32 (57.1%)
Time to engraftment^b^, median (range), days	16.9 (2–49)	16.8 (2–49)	17.7 (9–36)
*Antifungal prophylaxis*			
Fluconazole	441 (79.6%)	405 (81.3%)	36 (65.5%)
Micafungin	62 (11.2%)	52 (10.4%)	9 (16.4%)
mold-active	9 (1.6%)	6 (1.2%)	3 (5.5%)
Secondary	42 (7.6%)	35 (7.0%)	7 (12.7%)
*Acute GvHD*			
0–I	175 (65.8%)	159 (66.5%)	15 (57.7%)
II–IV	91 (34.2%)	80 (33.5%)	11 (42.3%)
*Chronic GvHD*			
absent/mild	166 (65.6%)	148 (65.5%)	17 (65.4%)
moderate/severe	87 (34.4%)	78 (34.5%)	9 (34.6%)
*Overall Survival*			
Alive	295 (52.4%)	275 (54.6%)	19 (32.8%)
Dead	268 (47.6%)	229 (45.4%)	39 (67.2%)

IFI, invasive fungal infection; AML, acute myeloid leukemia; MDS, myelodysplastic syndromes; ALL, acute lymphoblastic leukemia; HL, Hodgkin lymphoma; NHL, non-Hodgkin lymphoma; MPN, myeloproliferative neoplasms; LMMC, chronic myelomonocytic leukemia; HCT-CI, hematopoietic cell transplantation comorbidity index; DRI, disease risk index; CR, first complete remission; CR2, second complete remission; PIF, primary induction failure; MSD, matched sibling donor; MUD, matched unrelated donor; PBSC, peripheral blood stem cell; BM, bone marrow; CB, cord blood; IQR, interquartile range; MAC, myeloablative conditioning; RIC, reduced intensity conditioning; ATG, anti-thymocyte globulin; GvHD, graft-versus-host disease

a Including five patients who received HSCT from one antigen mismatched related donor.

b Engraftment is defined as the first of 3 days with neutrophils >0.5 × 10^9^/L after stem cell reinfusion.

Median age at the time of transplant was 48 years (range, 18–70) with 307 male (54.5%) and 256 female patients (45.5%).

Underlying diseases were acute leukemia (71.6%), Hodgkin and non-Hodgkin lymphomas (17.9%), chronic myeloproliferative disease (8.7%), and multiple myeloma (1.8%).

Slightly more than two-thirds of patients were transplanted in complete remission (282/563, 50.4% in first CR; 96/563, 17.1% in second CR) and 182 (32.5%) in advanced stage of disease. Patients received grafts from a matched sibling donor (191/563, 34%), from a matched unrelated donor (284/563, 50.5%), and from an haploidentical family member (87/563, 15.5%). Main graft source was peripheral blood in 81.5% of the patients.

### Cumulative Incidence and Characteristics of IFI

**Figure 1:**
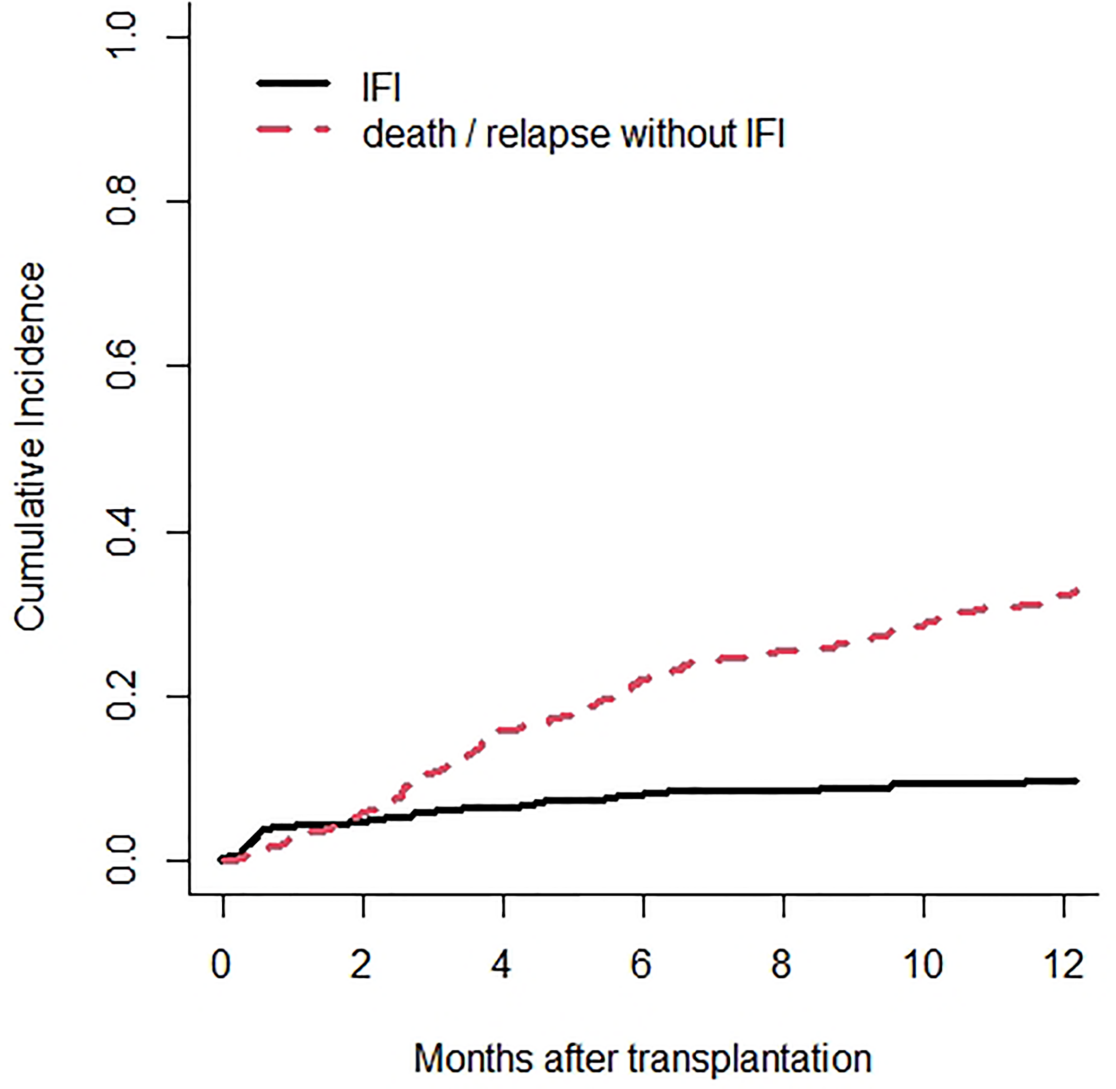
Cumulative incidence rate of invasive fungal infections (IFIs) is 9.6% at 12 months after transplant on the whole cohort. Death or relapse without IFIs are considered the competing event.

Overall, a PP-IFI was documented in 58 of 563 transplants (10.3%) resulting in a cumulative incidence of 4.1%, 8.1%, and 9.6% at 30, 180, and 365 days, respectively (**Figure 1**).

**Figure 2:**
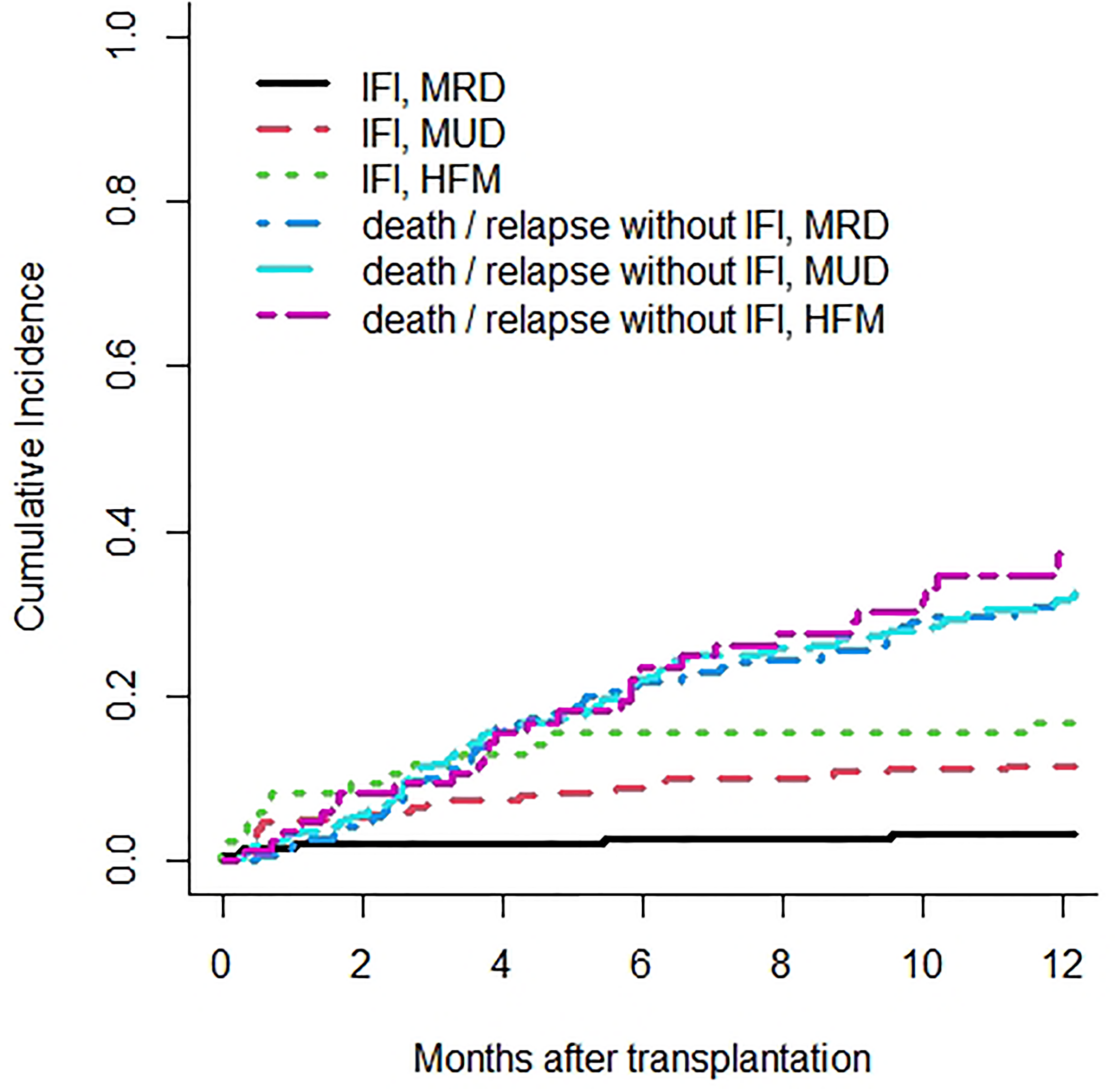
Cumulative incidence rates of invasive fungal infections (IFIs) are 3.2% in matched related donor (MRD), 11.4% in matched unrelated donor (MUD), and 16.8% in haploidentical family member (HFM) transplants. Death or relapse without IFIs are considered the competing event.

IFIs among different types of allogeneic HSCT had a 1-year cumulative incidence of 3.2% for matched related donor, 11.4% for matched unrelated donor, and 16.8% for haploidentical donor transplants (**Figure 2**).

Median time from HSCT to IFI diagnosis was 98.44 days, 56 days for candidiasis, and 104.38 days for mold infections.

Underlying diseases in patients who developed IFI were as follows: acute leukemia, 74.1%; HL/NHL, 15.5%; MPB/LMMC, 8.6%; MM, 1.7%. More than half of infections occurred in the post-engraftment phase (10/58—17.2% plus 22/58—37.9% in early and late post-engraftment phase, respectively), while 44.8% developed in the pre-engraftment period (26/58).

Among IFIs, molds were the predominant agents (n = 50 *Aspergillus*; n = 1 *Mucor*) and primarily involved the lung (n = 47; 92.2%), central nervous system (n = 2; 3.9%), bone (n = 1; 2%), and tonsils (n = 1; 2%). Invasive candidiasis occurred in seven patients (n = 5 non-*albicans Candida*; n = 1 C*. albicans*; n = 1 *Trichosporon*) and were bloodstream infections in all cases.

Eleven IFIs (19%) were proven infections, and 47 (81.0%) were classified as probable. Diagnosis in these latter group was based on a combination of imaging and positive galactomannan in serum (n = 24), BAL (n = 15), or both (n = 6), and in liquor (n = 2).

In the whole cohort, a total of 57 BALs were performed and were informative in a consistent number of cases (36/57, 63.2%) leading to the identification of fungal (21), bacterial (4), viral (3), and polymicrobial (8) infections (**Table 2**).

**Table 2:** Detailed report of pathogens detected by BAL in 36 patients.

Pathogen isolated	n.	%
**GM**	20	59
- Without fungal growth	19	
- With *Aspergillus fumigatus* growth	1	
**Microscoporon Rhizopus**	1	
**Bacteria**	4	11
- *P. aeruginosa*	2	
- *Haemophilus influenzae*	1	
- *E. coli* and *K. pneumoniae*	1	
**Viruses**	3	8
- Influenza B	1	
- Human parainfluenza virus	1	
- CMV, HHV-6, HHV-7	1	
**Polymicrobial**	8	22
- GM, HSV-1, *Bocavirus*	1	
- GM, RSV, *P. aeruginosa*	1	
- GM, RSV, *Staphylococcus aureus*	1	
- GM, *E. coli*	1	
- GM, *Mycobacterium fortuitum*	1	
- GM, *Mycobacterium avium*	1	
- GM, RSV	1	
- RSV, *K. pneumoniae*	1	

GM, galactomannan; P. aeruginosa, Pseudomonas aeruginosa; E. coli, Escherichia coli, K. pneumoniae, Klebsiella pneumoniae, CMV, cytomegalovirus; HHV-6, human herpesvirus 6, HHV-7, human herpesvirus 7; HSV-1, Herpes simplex virus 1; RSV, respiratory syncytial virus.

### Antifungal Practice

Primary antifungal prophylaxis (PAP) included fluconazole in 441 patients (79.6%) and micafungin in 62 (11.2%); only 9 patients received mold-active prophylaxis (1.6%). Overall, 79 patients (14.0%) received a diagnostic-driven treatment and 63 patients (11.2%) a fever-driven treatment. Liposomal Amphotericin-B (L-AmB) and voriconazole were the drugs used in the majority of patients receiving diagnostic-driven therapy (30/79, 38% and 22/79, 27.8%, respectively), while caspofungin was administered more frequently in patients who received a fever-driven strategy (27/63, 42.9%), followed by L-AmB (17/63, 27%) and voriconazole (11/63, 17.5%).

### Risk Factors for IFI

Univariate and multivariate competing risk regression model for IFIs occurrence are summarized in **Table 3**.

**Table 3:** Univariate and multivariate analysis of risk factors for invasive fungal infections after allogeneic HSCT.

	Univariate analysis		Multivariate analysis	
	SDHR *(*95% CI*)*	*p*	SDHR (95%CI)	*P*
Age (>60 vs. 40–60 vs. <40 years)	1.38 (0.95–2.01)	0.088	–	–
Gender (males vs. females)	0.94 (0.55–1.61)	0.820	–	–
Underlying disease (AML vs. other)	1.27 (0.73–2.22)	0.390	–	–
HCT-CI (≥3 vs. 0–2)	2.24 (1.02–4.92)	0.045	2.07 (0.93–4.57)	0.073
DRI (very high + high vs. intermediate vs. low)	1.29 (0.78–2.13)	0.320	–	–
Disease status at transplant (advanced vs. early disease)	1.49 (0.86–2.61)	0.160	–	–
Type of transplant (Haploidentical vs. MUD vs. MSD)	2.17 (1.51–3.11)	<0.001	1.91 (1,13–3.20)	0.015
Graft source (PBSC vs. BM)	0.71 (0.37–1.39)	0.320	–	–
Number of CD34+ cells infused (over vs. under median), 10^6^/kg	0.71 (0.40–1.26)	0.240	–	–
Number of CD3+ cells infused (over vs. under median), 10^8^/kg	0.89 (0.49–1.61)	0.690	–	–
Conditioning (RIC vs. MAC)	1.60 (0.92–2.78)	0.098	–	–
GvHD prophylaxis (ATG vs. other)	1.53 (0.87–2.68)	0.140	–	–
Time to engraftment (over vs. under median), days	1.54 (0.85–2.78)	0.150	–	–
Antifungal prophylaxis (micafungin vs. fluconazole)	2.06 (0.99–4.30)	0.054	–	–
Acute GvHD II–IV (yes vs. no)	1.49 (0.68–3.26)	0.320	–	–
Chronic GvHD moderate–severe (yes vs. no)	1.05 (0.47–2.35)	0.910	–	–

SDHR, subdistribution hazard ratio; CI, confidence interval; AML, acute myeloid leukemia; HCT-CI, hematopoietic cell transplantation comorbidity index; DRI, disease risk index; MUD, matched unrelated donor; MSD, matched sibling donor; PBSC, peripheral blood stem cell; BM, bone marrow; RIC, reduced intensity conditioning; MAC, myeloablative conditioning; ATG, anti-thymocyte globulin; GvHD, graft-versus-host disease.

A high HCT-CI (≥3), the donor type (haploidentical vs. MUD vs. MRD), and the use of micafungin as antifungal prophylaxis were associate with a higher risk for IFI [subdistribution hazard ratio (SDHR), 2.24, *p* = 0.045; SDHR, 2.17, *p* < 0.001; SDHR, 2.06, *p =* 0.054, respectively]. Only the donor type reached significance in the multivariate model (SDHR, 1.91; *p* = 0.015).

### Outcome and Mortality

The median follow-up of surviving patients undergoing allogeneic HSCT during our study period was 6.70 years (0.14–17.02), in particular 5.68 (range, 0.28–15.48) years for patients with IFI and 6.79 (range, 0.14–17.02) years for patients without IFI.

Overall, relapse was the major cause of death (168/268, 62.7%), while 100 patients died of transplant-related complications (100/268, 37.3%). Nine patients with PP-aspergillosis (18%), 1 patient with invasive candidiasis (16.7%), and the patient with mucormycosis died of infection. IFI-related mortality rate was 20.7% in the overall population, 17% in patients with probable IFI, and 36% in patients with proven IFI.

**Figure 3:**
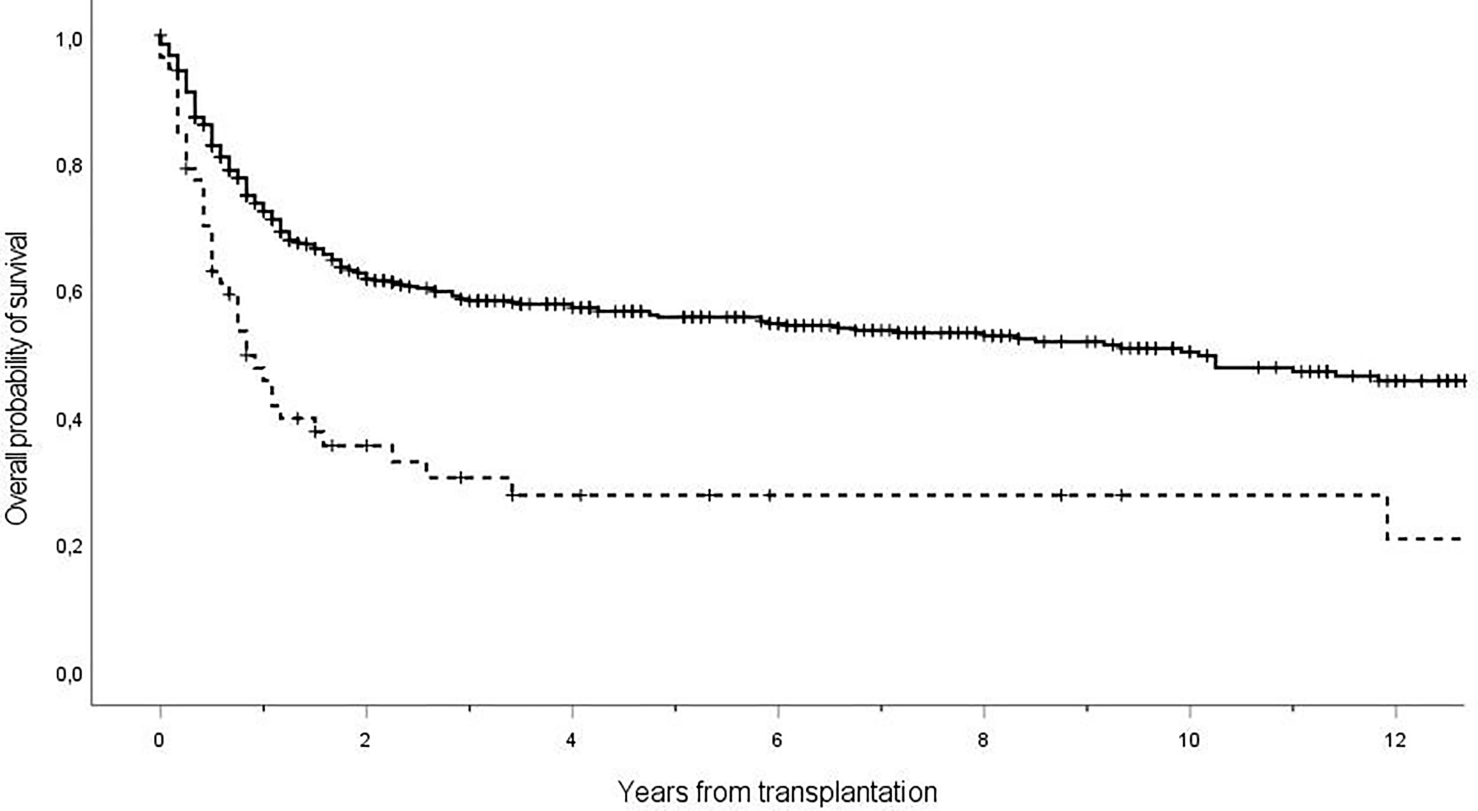
Overall survival of patients receiving allogeneic transplant with diagnosis of IFI (dashed line) or not (solid line). The 1-year overall survival was 32.8% in patients IFI positive vs. 54.6% in patients IFI negative, with a statistically significant difference (*p* < 0.001). Ticks on probability lines indicate dates of censoring at last follow-up.

The presence of IFI had a significant impact on overall survival at 1 year (IFI, 32.8% vs. non-IFI, 54.6%; *p* < 0.001) (**Figure 3**).

## Discussion

In the present study, we evaluated the epidemiology and the prognostic factors of IFIs in a cohort of HSCT patients receiving PAP mainly with fluconazole and a diagnostic-driven therapy guided by screening with NCBM, BAL, and CT scan.

Overall, the cumulative incidence of PP-IFI at 1-year post-HSCT was higher as compared to the TRANSNET (Kontoyiannis et al., 2010) and SEIFEM (Pagano et al., 2007) trials (9.6% vs. 3.4% and 3.7%, respectively) but superimposable to the 7–9% rates reported in the most recent studies (Girmenia et al., 2014; Van de Peppel et al., 2018).

According to a consistent number of studies evaluating the epidemiology of IFI in hematological patients [TRANSNET (Kontoyiannis et al., 2010), SEIFEM (Pagano et al., 2007), and PATH (Neofytos et al., 2009)], invasive aspergillosis was the most predominant IFI accounting for three quarters of the infections. Non-*albicans* spp. were the most frequent yeast infection, underpinning that the generalized use of azoles may induce a selection pressure in favor of non-*albicans* spp.

According to ECIL guidelines (Maertens et al., 2018), fluconazole may be considered for PAP during the pre-engraftment phase when an intensive diagnostic work-up is part of the strategies for management of febrile patients. In our study, the median time onset of IFI was largely beyond the engraftment phase. Nevertheless, the presence of neutropenia at the time of IFI was detected in 34.5% of the patients, supporting the observation that delayed neutropenia is a frequent complication in HSCT recipients [in particular, MUD and haploidentical HSCT recipients (Di Stasi et al., 2014; Salvatore et al., 2018)] usually related to concomitant treatments for cytomegalovirus (CMV) infections and GvHD. In this respect, we have implemented our policy to include mold-active prophylaxis in patients with late cytopenias or GvHD requiring systemic immunosuppressive treatments.

In line with these observations, the high rate of IFI observed in our series of MUD and haploidentical recipients might be explained with the increased risk of viral infections and GvHD reported in many studies (Dominietto et al., 2001).

As reported in a previous study (Busca et al., 2018), a high HCT-CI (≥3) was predictive of the risk of IFIs in univariate analysis and resulted in marginal significance in the multivariate model. If prospective trials will confirm this observation, patients with high HCT-CI at the time of transplant might benefit from a more aggressive strategy including antifungal prophylaxis with mold-active agents.

The lung was the most frequent site involved in patients with mold infections. Notably, 51% of our patients with pulmonary infiltrates were explored with BAL, supporting the concept that BAL is a diagnostic procedure feasible in a consistent number of hematological patients. Even more importantly, BAL led to the identification of the causative agent in two-thirds of the cases. Our data compare favorably with the study published by the SEIFEM group, showing that 33% of patients with lung infiltrates were investigated, with BAL yielding the identification of the putative microbiological agent in 75% of the cases (Marchesi et al., 2019).

Mold-active agents include azoles (i.e., itraconazole, voriconazole, Posaconazole, and isavuconazole) targeting the fungal lanosterol 14α-demethylase, leading to an alteration in cell membrane permeability, and polyenes (i.e., amphotericin B) binding to the membrane ergosterol with consequent formation of pores and channels that irreversibly damage the fungal wall. Echinocandins represent the most effective agents for yeast infections by the inhibition of the synthesis of 1,3-β-D-glucan, which is a major component of the cell wall.

Despite the use of a non-mold-active agent as antifungal prophylaxis, our study showed that a therapeutic approach driven by a diagnostic strategy including NCBM, BAL, and CT scan is effective, resulting in a low mortality rate (21%) while avoiding unnecessary toxicity. Even in patients with proven IFI, the mortality was 36% significantly lower when compared to the 72% mortality rate reported in a recent meta-analysis of Van Peppel et al. (2018). It should be emphasized that the availability of a wide armamentarium of antifungal agents with different spectrum of activity and good safety profiles may have contributed to improve the outcome of patients with IFI. Nevertheless, it should be emphasized that the presence of IFIs had a remarkable impact on the overall survival (IFI, 32.8% vs. non-IFI, 54.6%; p < 0.001). In this respect, antifungal stewardship in hematological patients is eagerly needed: new diagnostic tools able to anticipate the identifications of patients with IFI and prognostic risk score that might predict the risk of IFI may represent resources of utmost importance.

In total, 11% of our patients received a fever-driven treatment. This negligible number of empirical treatments demonstrates that the application of an intensive work-up is able to limit the use of unnecessary treatments, even in HSCT recipients generally deemed as critical ill patients. Among these patients, 11 (17.5%) died of infection.

Several limitations of our analysis should be acknowledged. The study is retrospective and is a single-center study: accordingly, the epidemiology might not be representative of the spectrum of the fungi distributed in different transplant centers. Regrettably, the low number of proven mold infections did not allow us to investigate the possible emergence of azole resistance as described in other studies (Shariati et al., 2020).

In summary, the results of our study indicate that febrile patients after HSCT may benefit from a comprehensive diagnostic work-up including NCBM, early CT scan, and BAL. This approach unveiling a low mortality rate may be relevant to determine additional interventions aiming to improve the outcome of patients who develop IFI.

## Data Availability Statement

The raw data supporting the conclusions of this article will be made available by the authors, without undue reservation.

## Ethics Statement

All procedures performed in the present study were in accordance with the ethical standards of the institutional and/or national research committee and with the 1964 Helsinki declaration and its later amendments or comparable ethical standards.

## Author Contributions

All authors listed have made a substantial, direct, and intellectual contribution to the work and approved it for publication.

## Conflict of Interest

AB has received honoraria from Gilead Sciences, Merck, Pfizer Pharmaceuticals, and Jazz Pharmaceuticals; he has been a speaker for Gilead Sciences, Merck, Pfizer Pharmaceuticals, and Novartis; and he is part of an Advisory Board of GILEAD and Pfizer. LG has been a speaker for Pfizer, Jazz, and Novartis.

The remaining authors declare that the research was conducted in the absence of any commercial or financial relationships that could be construed as a potential conflict of interest.

## Publisher’s Note

All claims expressed in this article are solely those of the authors and do not necessarily represent those of their affiliated organizations, or those of the publisher, the editors and the reviewers. Any product that may be evaluated in this article, or claim that may be made by its manufacturer, is not guaranteed or endorsed by the publisher.
